# Enlargement of the Prostate

**Published:** 1865-04

**Authors:** F. R. Payne

**Affiliations:** Marshall, Illinois


ARTICLE XIII.

### ENLARGEMENT OF THE PROSTATE.

BY F. R. PAYNE, M.D., Marshall, Ill.

The prostate is not unfrequently enlarged by acute inflammation from virulent gonorrhœa, blows inflicted on the perineum, or the rough and improper use of the catheter. There are other causes capable of producing this disease. In acute inflammation of this gland the symptoms are positive, and with proper care in the examination it is readily detected. If true inflammation continues for any considerable time, we frequently have abscess of the gland. When this occurs, the prompt resort to the lancet or trocar is the most reliable hope for the prevention of urinary abscesses and recto-vesical fistula. We sometimes find chronic suppuration of this gland, and when this occurs there is much distress, and frequent discharge of muco-purulent urine. This can usually be detected by introducing the finger into the rectum. If a soft point is found slightly elevated, and, by pressing, matter escapes through the urethra, we are justifiable in plunging the lancet or trocar, and thus remove the pus, give immediate relief, and save the patient from the horrors of an offensive and troublesome fistula.

My object in writing this paper is to present to your readers a brief history of one case which recently occurred in this locality.

Mr. James C. Graham, aged 68 years, a farmer by occupation, a virtuous and respectable citizen, sent for me on the 23d day of March, 1865, and I found him laboring under the following symptoms:—Excessive pain in the back and urinary organs; complete suppression of urine, and extreme constipation, with almost a constant desire to evacuate the contents of the bowels and bladder; very restless and nervous; but very little arterial excitement; pulse intermittent; tongue coated with a brown fur; breath offensive; a fulness of the epigastrium; nausea, and occasional efforts at vomiting. For the last eight or ten years he has had great trouble in voiding urine, and four years ago he had an abscess, which was discharged through the rectum, relief immediately followed, without the troublesome effects of fistula. For several weeks previous to the time we saw him, he had suffered greatly with the symptoms above enumerated, but did not call the aid of a physician until after the complete suppression of urine. We visited him early in the morning, three miles in the country, and made an effort to introduce the catheter, no obstacle was encountered until the point of the instrument reached the upper portion of the prostate. After cautious, and more than ordinary perseverance, we failed to accomplish our object. I then made the following prescription:—

| | | |
|---|---|---|
| ℞. | Hydrg. Chld. Mit. | 50 grs. |
| | Pulv. Opi. | 6 grs. |
| | Pulv. Ipecac | 15 grs. |

Mix, and make three powders. Ordered one every two hours; in five hours after last, if no operation, oil, or seidlitz; also, free use of the tea of buchu leaves.

*Evening.*—No operation from bowels or bladder. Another effort was made to introduce catheter, it seemed surely to pass into the organ, but only a few tablespoonfuls of thick, bloody matter was discharged, and that only when the patient made an effort to void urine. But little pain attended the operation. Ordered hip bath, laxatives, and rectal injections.

*24th.*—No fecal or urinary discharges; patient very restless, sick at stomach, and anxious expression of the countenance; pulse 90, and intermittent. Catheter again introduced, only resulting in the discharge of one or two ounces of bloody, thick material. We then passed a tube of stomach-pump up to sygmoid flexion, and through it large enemata were used. This did no good, the injections would pass out as though they were driven back with a force-pump. Fomentations were then ordered over the hypogastric region, sinapisms to spine, calomel and opium internally. If this failed, four drops of oleum tiglii every hour, in four doses.

*25th.*—Patient still not relieved; no action from the medicine ; pulse slow, but intermittent, indicating some toxic agent in the system. Persistent efforts were still made to procure an evacuation from the kidneys and bowels, but failed, and he died in the later part of the day in great agony.

*Post Mortem.*—On the 26th, Drs. GEORGE F. CENTER, HENRY P. BROOKHEART, and myself, opened the abdomen and found extensive inflammation of the lining membranes of the bladder. That organ was considerably distended with a thick bloody material resembling that discharged through the catheter. The ascending, and part of the transverse colon, above the point of pressure of the enlarged prostate, was highly inflamed, below that point the ascending colon and rectum was healthy. The gland when removed, after being thoroughly cleansed, weighed five ounces and five drachms. When cut through, many evidences are presented of previous suppuration.

